# Detection of *Galba truncatula*, *Fasciola hepatica* and *Calicophoron daubneyi* environmental DNA within water sources on pasture land, a future tool for fluke control?

**DOI:** 10.1186/s13071-018-2928-z

**Published:** 2018-06-08

**Authors:** Rhys Aled Jones, Peter M. Brophy, Chelsea N. Davis, Teri E. Davies, Holly Emberson, Pauline Rees Stevens, Hefin Wyn Williams

**Affiliations:** Institute of Biological, Environmental and Rural Sciences (IBERS), Aberystwyth University, Aberystwyth, Ceredigion Wales, UK

**Keywords:** *Galba truncatula*, *Fasciola hepatica*, *Calicophoron daubneyi*, Environmental DNA, Trematode, Snail

## Abstract

**Background:**

Increasing trematode prevalence and disease occurrence in livestock is a major concern. With the global spread of anthelmintic resistant trematodes, future control strategies must incorporate approaches focusing on avoidance of infection. The reliance of trematodes on intermediate snail hosts to successfully complete their life-cycle means livestock infections are linked to the availability of respective snail populations. By identifying intermediate snail host habitats, infection risk models may be strengthened whilst farmers may confidently apply pasture management strategies to disrupt the trematode life-cycle. However, accurately identifying and mapping these risk areas is challenging.

**Methods:**

In this study, environmental DNA (eDNA) assays were designed to reveal *Galba truncatula*, *Fasciola hepatica* and *Calicophoron daubneyi* presence within water sources on pasture land. eDNA was captured using a filter-based protocol, with DNA extracted using the DNeasy® PowerSoil® kit and amplified *via* PCR. In total, 19 potential *G. truncatula* habitats were analysed on four farms grazed by livestock infected with both *F. hepatica* and *C. daubneyi*.

**Results:**

*Galba truncatula* eDNA was identified in 10/10 habitats where the snail was detected by eye. *Galba truncatula* eDNA was also identified in four further habitats where the snail was not physically detected. *Fasciola hepatica* and *C. daubneyi* eDNA was also identified in 5/19 and 8/19 habitats, respectively.

**Conclusions:**

This study demonstrated that eDNA assays have the capabilities of detecting *G. truncatula*, *F. hepatica* and *C. daubneyi* DNA in the environment. Further assay development will be required for a field test capable of identifying and quantifying *F. hepatica* and *C. daubneyi* infection risk areas, to support future control strategies. An eDNA test would also be a powerful new tool for epidemiological investigations of parasite infections on farms.

## Background

Trematode parasites are amongst the most important pathogenic organisms of livestock worldwide. Among the trematode family, the liver fluke (*Fasciola hepatica*) infects livestock on six continents, with its disease (fasciolosis) estimated to cost the global livestock production industry €2.5 billion annually [1]. Other trematode parasites such as rumen flukes (*Calicophoron daubneyi* and *Paramphistomum* spp.), have emerged as parasites of concern in numerous temperate countries over the past decade [2–4] with climate change likely fuelling increasing trematode prevalence and disease occurrence in livestock worldwide [5, 6]. In the continued absence of vaccines, trematodosis control in livestock is mostly *via* anthelmintic administration, however the sustainability of this practice is under threat due to the spread of anthelmintic resistance and limited evidence of new flukicide compounds [7].

Future control for trematodes should incorporate strategies focusing on avoidance of infection [7, 8]. Clearly, as both *F. hepatica* and *C. daubneyi* are reliant on the same intermediate snail host, *Galba truncatula* [9], infection risk areas for these parasites will be limited to pastures harbouring these snails. *Galba truncatula* snails are highly dependent on wet climatic and environmental conditions [10], and their presence and densities will vary within fields and thus, specific fluke infection risk areas will be present on farms [11]. Identifying these risk areas will strengthen trematode infection risk models [12], and support farmers to implement farm management practices such as drainage, rotational grazing and fencing and thereby reduce contact between vulnerable animals and the infective metacercariae. Obviously, fluke infection risk areas must first be accurately identified to maximise the cost-benefit of these often expensive practises.

The traditional method of physically identifying *G. truncatula* habitats by eye remains labour intensive, time consuming and is subsequently costly [12]. Novel techniques incorporating satellite imagery and GIS have recently been developed to identify potential *G. truncatula* habitats [13, 14]. However, these cannot identify the presence or intensity of snails within identified habitats. This is a major issue considering that a high percentage of seemingly suitable *G. truncatula* habitats may be uninhabited by the snail due to variations in habitat suitability arising due to pasture management or its environmental features [12, 15]. In addition, even in instances where *G. truncatula* snails are correctly identified within a habitat, their infection status will be unknown and may vary between habitats due to their previous exposure to miracidial stages of the fluke parasites and their susceptibility to parasitic infections [16].

Considering that *G. truncatula* snails and external infective stages of *F. hepatica* and *C. daubneyi* are predominantly found in aquatic environments, water-based environmental DNA (eDNA) assays may be a valuable tool for identifying the presence of both snail and digenean species, and subsequently to estimate the infection risk for livestock grazing a pasture. New eDNA detection techniques are increasingly being developed, optimized and adopted by researchers across a large array of fields to identify and monitor the presence of animal, plant, fungal, protist and prokaryote organisms in the environment [17]. These techniques seek to detect DNA sourced from excreted cells and tissues including skin, hairs, mucus, faeces and urine in environmental samples such as freshwater, seawater, sediments, soil and ice [17], and are regarded as having enormous potential for monitoring disease risk [18]. In recent years, eDNA techniques have been successfully developed and optimised to detect the presence of *Potamopyrgus antipodarum* [19] and *Physa acuta* snails among others [20], which suggests that the technique may be applied for other snail species, including *G. truncatula.* eDNA techniques have also been used within the field of parasitology, where eDNA originating from the microscopic external stages of trematode species, *Ribeiroia ondatrae* and *Opisthorchis viverrini* have been detected in freshwater [18, 21].

In this study, an eDNA assay was developed to detect the presence of *G. truncatula* snails in areas of pasture land. The assay was further developed to identify the presence of *F. hepatica* and *C. daubneyi* larval stages within water sources on pasture land. These assays were tested on commercial livestock farms with the aim of confirming that DNA from these organisms was present and detectable within the environment.

## Methods

### eDNA capture and extraction

A filter-based eDNA capture and extraction protocol was used throughout this study. Water samples were filtered through glass microfiber filters using a vacuum filtration kit. During this process, a filter was placed in a 5 cm funnel attached to a 500 ml Buchner flask. A brake bleeding pump was used to create a vacuum within the Buchner flask, leading to the flow of water through the filter where DNA strands from the water sample were concentrated. After each filtration, the filter holder, scissors (used to cut the filter) and tweezers (used to handle filters) were soaked in 10% bleach for 5 min to avoid cross-contamination between samples. DNA was extracted from filter samples using the DNeasy® PowerSoil® kit (Qiagen, Venlo, Netherlands). Each filter, including blank controls, was homogenised using a sterile pipette tip and approximately a quarter of the filter was subjected to DNA extraction *via* the PowerSoil® kit protocol. A quarter of the filter was extracted as this was the maximum that could be processed within a single PowerSoil® kit extraction tube.

### eDNA laboratory experiments

Laboratory experiments tested two hypotheses: (i) that *G. truncatula* and digenean DNA would be detectable in water previously harbouring each organism; and (ii) that eDNA from these organisms could be concentrated on 2.7 μm pore size glass microfiber filters. Five *G. truncatula* snails, collected from the field, were placed for 12 h in each of four plastic containers. Each container was filled with 1 l of double distilled water. A total of 500 ml of water from each experimental container was filtered through 5 cm 2.7 μm pore size glass microfiber filters (GF/D) (Whatman plc, Maidstone, UK) at day 1, 4, 7 and 14 post-snail removal, respectively. This filtered water was then immediately re-filtered through 5 cm 0.7 μm pore size glass microfiber filters (GF/F) (Whatman plc, Maidstone, UK) to test if target eDNA could escape through the larger pore sized filter. Filters were cut in half with sterile scissors and stored in 2 ml centrifuge tubes at -20 °C prior to DNA extraction.

An almost identical protocol was followed for the digenean eDNA experiment. Here, ten newly hatched *C. daubneyi* miracidia were placed in 1 l of double distilled water within each of four plastic containers. Water was filtered and re filtered as described above at day 1, 4, 7 and 14 post-miracidial death. Parallel to this experiment, *C. daubneyi* miracidia were placed in water within a 5 cm Petri dish and their survival and tissue degradation rates at room temperature were monitored during the experimental period.

### Field sampling protocol

To validate the success of the eDNA assays in identifying the presence of *G. truncatula*, *C. daubneyi* and *F. hepatica* on pasture land, water samples were taken from habitats identified to be potentially suitable for *G. truncatula* habitation on four Welsh farms known to have ruminants infected with both *C. daubneyi* and *F. hepatica*. These habitats included poached grassland, drainage ditches and ponds, all of which contained standing or slow-moving freshwater. Samples were taken on three visits in June, July/August and September 2017. *Galba truncatula* snails were collected where present from these habitats following a 10 min search and screened for parasitic infections using microscopy and polymerase chain reaction (PCR) methods as described by Jones et al. [9]. Mob faecal samples from each sheep flock and cattle herd grazing each farm were also collected and subjected to sedimentary FEC to identify if livestock on respective farms were shedding fluke eggs *via* the methods described by Jones et al. [4].

A total of 500 ml water from the habitats was filtered through 5 cm 2.7 μm pore size glass microfiber filters (GF/D) from each potential *G. truncatula* habitat during each visit. This filter pore size was selected after the laboratory testing showed that loss of eDNA through this filter was minimal (see results section) and it afforded the larger water filtering capacity compared to smaller pore size filters [22]. This 500 ml water sample was collected from 10 random locations (50 ml × 10) within the sampled area. Whilst sampling, care was taken to avoid disturbing the sediment in sampled water bodies as excessive humic substances in a sample would rapidly clog the filter. Following filtration, the filter was cut in half with scissors and then submerged in 500 μl of CTAB buffer (10%). The samples were stored at room temperature as described by Renshaw et al. [23]. This storage method has been shown to preserve DNA on polycarbonate track etch (PCTE) filters for 150 days [24].

### PCR

Three PCR assays were designed to amplify eDNA extracted from filtered water samples. Each PCR assay was optimised to amplify species-specific *G. truncatula*, *F. hepatica* and *C. daubneyi* DNA sequences, respectively. As DNA is known to degrade rapidly in the environment, only short strands of eDNA may be present in water [18]. Thus, to increase eDNA PCR sensitivity, only primers amplifying DNA strands less than 300 bp in length were used [25]. The *ITS2* gene was chosen as the target gene for all eDNA PCR as the *ITS2* sequence is known to occur in tandem repeats of often thousands of copies within the ribosomal DNA [26]. Thus, these sequences may be present in larger quantities within the environment compared to sequences from other genes [25]. Primers were designed using the Geneious software [27]. During this process, DNA sequences within a gene of interest from the target species and other related trematode species attained from the National Centre for Biotechnology Information (NCBI) database were aligned. Unique DNA sequences within each respective species’ selected gene were identified and tested for their suitability as individual and paired primers using Primer3Plus software [28]. Designed primer sequences were cross-referenced with NCBI sequences *via* primer-BLAST to ensure species specificity to this study’s target eDNA. Primers (F: 5'-GTG AGC TCT CAC GCT GCT C-3' and R: 5'-TAG AGC CCC TTG TTC TCC A-3') were designed and used to amplify 288 bp *G. truncatula* eDNA; primers (F: 5'-CCC CTA GTC GGC ACA CTT A-3'; and R: 5'-TAT GAA ANT AGC ATC AGA CAC ATG A-3') were designed and used to amplify 169 bp *F. hepatica* eDNA; and primers (F: 5'-GGG TGT GGC GGT AGA GTC-3'; R: 5'-CGG ACR GCA ATA GCA TCT CAA-3') were designed and used to amplify 100 bp rumen fluke species eDNA.

For each PCR reaction, a 25 μl master mix was created containing 12.5 μl of Platinum™ Green Hot Start PCR Master Mix (ThermoFisher, Hayward, USA), 0.5 μl of each 10 μM primer, 2 μl of the extracted DNA and nuclease-free water. Each sample was subjected to PCR amplification consisting of an initial denaturation at 95 °C for 2 min followed by 40 cycles consisting of stages of denaturation (30 s at 95 °C) annealing (30 s at 61 °C) and extension (60 s at 72 °C), before a final 10 min extension phase at 72 °C. PCR products were visualised in 2% agarose gel stained with GelRed (Biotium, Hayward, USA) along with positive and negative controls. Subsets of PCR amplicons were sequenced to validate the specificity of the eDNA identified *via* PCR. PCR products were subjected to clean up using the PureLink® PCR purification kit (Fisher Scientific, Waltham, USA), prior to sequencing using an ABI Prism® 3100 DNA analyser (Applied Biosystems, Waltham, USA). Sequences were aligned to each specific species’ reference sequence on GenBank using Geneious software [27].

## Results

### eDNA laboratory experiment

*Galba truncatula* and *C. daubneyi* DNA was successfully extracted and amplified from water samples in the laboratory experiments (Table 1) indicating that extracellular DNA from these species may be present and detectable in environmental samples. *Calicophoron daubneyi* miracidial DNA was detectable in water following full visible degradation, which had occurred within 3 days of death. This eDNA, along with *G. truncatula* eDNA was shown to be capable of remaining detectable in the water for 7 days after death and removal, respectively. Detectable *G. truncatula* and *C. daubneyi* DNA was shown to flow through 2.7 μm pore sized glass microfiber filter, however, in each instance where DNA was amplified from 0.7 μm pore sized filters, eDNA was also amplified from the 2.7 μm pore sized filters.

**Table 1:** eDNA extraction and detection in laboratory experiments

DNA source	Filter (μm)	Day 1	Day 4	Day 7	Day 14
*G. truncatula* (*n* = 5)	2.7	+	+	+	-
	0.7	+	-	-	-
*C. daubneyi* miracidia (*n* = 10)	2.7	+	+	+	-
	0.7	+	+	-	-

*Key*: +, positive, -, negative

### eDNA field survey

Nineteen potential *G. truncatula* habitats were identified and sampled for eDNA on four Welsh farms (Table 2). *Galba truncatula* snails were identified and collected in ten of these habitats. *Galba truncatula* eDNA was amplified from water originating from each of these habitats (10/10 habitats) during one or more of the sampling visits. *Galba truncatula* eDNA was also amplified from water originating from four habitats where no *G. truncatula* snails were visually recorded. Sequencing of *G. truncatula* eDNA amplicons from each positive habitat (*n* = 14) confirmed primer specificity as all amplicons sequenced showed > 99% identity with the *ITS2* reference sequence (GenBank: AJ296271.1).

**Table 2:** *Galba truncatula* eDNA detection in habitats on four Welsh farms

Habitat^a^	Visit 1		Visit 2		Visit 3	
	GT density	GT eDNA	GT density	GT eDNA	GT density	GT eDNA
Confirmed *G. truncatula* habitats^b^						
A1	High	+	Absent	-	Absent	-
A2	Absent	+	Low	-	Absent	-
B1	Low	-	Absent	+	Absent	-
B2	Low	+	Absent	+	Absent	-
B3	Low	+	Absent	+	Absent	-
C1	Low	+	Low	-	Low	+
C2	Low	+	Medium	-	Medium	+
C3	Medium	+	Absent	+	Absent	+
D1	Low	+	Absent	+	High	+
D2	Low	+	Absent	-	High	+
Unconfirmed *G. truncatula* habitats						
A3	Absent	+	Absent	-	Absent	-
A4	Absent	-	Absent	-	Absent	-
A5	Absent	-	Absent	-	Absent	+
A6	Absent	-	Absent	-	Absent	-
A7	Absent	-	Absent	-	Absent	-
A8	Absent	-	Absent	+	Absent	-
B4	Absent	-	Absent	-	Absent	-
B5	Absent	-	Absent	-	Absent	-
C4	Absent	+	Absent	-	Absent	-

*Abbreviations*: *GT G. truncatula*; +, positive; -, negative

^a^Letter indicates farm; number indicates habitat within farm

^b^Low density: < 5 GT snails collected per visit; medium density: 5–15 GT; high density: > 15 GT

*Fasciola hepatica* eDNA was amplified from water originating from five habitats, three of which contained *G. truncatula* snails infected with *F. hepatica* (Table 3)*.* Sequencing of *F. hepatica* eDNA amplicons from each positive habitat (*n* = 5) confirmed primer specificity as all amplicons sequenced showed > 99% identity with the *ITS2* reference sequence (GenBank: AJ272053.1).

**Table 3:** *Fasciola hepatica* and *C. daubneyi* eDNA detection in habitats on four Welsh farms

Habitat^a^	FH infections in GT	CD infections in GT	Visit 1		Visit 2		Visit 3	
			FH eDNA	CD eDNA	FH eDNA	CD eDNA	FH eDNA	CD eDNA
Confirmed *G. truncatula* habitats								
A1	Yes	No	-	-	-	-	-	-
A2	No	No	-	+	-	-	-	-
B1	No	No	-	-	-	-	-	-
B2	No	No	-	-	-	+	-	-
B3	No	No	-	-	-	-	-	-
C1	Yes	Yes	+	-	-	-	-	-
C2	Yes	Yes	+	+	-	-	-	-
C3	Yes	Yes	+	+	-	-	+	-
D1	No	No	+	-	-	-	-	-
D2	No	No	-	-	-	-	-	-
Unconfirmed *G. truncatula* habitats								
A3	na	na	-	-	-	-	-	-
A4	na	na	-	+	-	-	-	-
A5	na	na	-	+	-	-	-	-
A6	na	na	-	-	-	-	+	-
A7	na	na	-	-	-	-	-	-
A8	na	na	-	-	-	+	-	-
B4	na	na	-	-	-	+	-	-
B5	na	na	-	-	-	-	-	-
C4	na	na	-	-	-	-	-	-

*Abbreviations*: *GT G. truncatula*, *FH* F. hepatica, *CD* C. daubneyi

^a^Letter indicates farm; number indicates habitat within farm

Rumen fluke eDNA was amplified from water originating from eight habitats, two of which contained *G. truncatula* snails infected with *C. daubneyi* (Table 3). Sequencing of each rumen fluke eDNA amplicon (*n* = 8) confirmed that the eDNA of *C. daubneyi* was present in each sample as all amplicons sequenced showed > 99% identity with its *ITS2* reference sequence (GenBank: AB042188.1).

## Discussion

This paper demonstrates that eDNA assays have the capability of detecting *G. truncatula*, *F. hepatica* and *C. daubneyi* eDNA in water collected from pasture land. Overall, the assay detected *G. truncatula* DNA in 100% of habitats proven to harbour the snail by eye (*n* = 10). *Galba truncatula* eDNA was also identified in four habitats where *G. truncatula* snails were not physically observed, indicating the assay has the potential to be more sensitive than the traditional method of detecting *G. truncatula* habitats by eye. Accurately identifying *G. truncatula* habitats using traditional methods may be difficult due to the variable nature of *G. truncatula* populations, which lead to short term variations in the snails’ visible presence and population size [10, 11]. Specialist training is therefore required to identify potential habitats and to detect and differentiate between *G. truncatula* and other similar snail species commonly observed in overlapping niches [29]. This process is impractical for farmers, whilst hiring experts in this area may be costly, even if their expertise could be supplemented with novel habitat detection methods such as GIS and satellite technology. With further refinement, an eDNA kit could offer a rapid, cheap and reliable method of identifying *G. truncatula* habitats in the future. This could help farmers, veterinarians and researchers to identify fluke infection risk areas on pasture land and develop integrated control strategies that are less reliant on anthelmintics.

*Fasciola hepatica* and *C. daubneyi* eDNA was also detected in water samples collected from pasture land in this study, however, interpreting digenean eDNA detection in pasture sourced water is complex as various free-living infective and non-infective digenean stages may be the source of eDNA. Both *F. hepatica* and *C. daubneyi* eDNA was amplified from habitats where *G. truncatula* snails were not detected over three visits. In confirmed *G. truncatula* habitats, *F. hepatica* and *C. daubneyi* eDNA was amplified from samples collected in June, a period which is not traditionally associated with large numbers of cercariae and metacercariae being present in the study area [30]. These findings would indicate that the main source of amplified *F. hepatica* and *C. daubneyi* eDNA may be miracidia, which would likely have been abundant on sampled farms as liver and rumen fluke eggs were detected in livestock faeces throughout the study period. Laboratory-based experiments demonstrated that the eDNA assay could amplify *C. daubneyi* DNA from fully degraded miracidia in water. Any miracidia failing to infect an intermediate host, a frequent occurrence [31], are therefore likely to release DNA into water which may subsequently be detected. It remains unclear whether other digenean stages are major sources of eDNA. Cercariae are a likely source of eDNA, although their transformation into metacercariae may only take a few hours [32] and thus, the timeframe for their potential capture will be short. However, other sources of cercarial eDNA may include their tail, which is shed during encystment [33], and dead cercariae, a common occurrence for cercariae shed from snails due to increased risk of tissue rupture [32]. Unhatched eggs and metacercariae are unlikely to release significant amounts of DNA into the environment. Metacercarial DNA is enclosed by a protective layered shell [34, 35] which protects against desiccation, mechanical injury and toxic substances [36], whilst it has been shown that the shell of *Fasciola* spp. eggs must be mechanically disturbed for it to release DNA [37]. Further laboratory experiments to quantify the amount of eDNA released by all free-living digenean stages will be needed to be able to fully interpret what stages of digeneans are associated with eDNA being present in pasture water.

The problematic differentiation between the non-infective miracidial and infective cercarial stages may not be a major limitation for application of eDNA assays for fluke control on farms. For example, analysis by Jones et al. [9] demonstrated that increasing numbers of liver and rumen fluke eggs shed onto a habitat was significantly associated with increasing infection prevalence of each respective fluke within *G. truncatula* populations. This indicates that in the presence of *G. truncatula* snails and ruminants, the detection of *F. hepatica* and *C. daubneyi* eDNA in pasture water would signal that the life-cycles of each parasite are likely to be active in the area. Further transcriptome analysis of digeneans and developments in environmental RNA (eRNA) detection in the future may enable assays to be created which may differentiate between key parasite stages by amplifying RNA from genes upregulated during a specific stage of a digenean’s life-cycle [18]. These developments would allow eRNA assays to monitor levels of cercariae released from snails leading to estimations of short term infection risk on pasture. As of yet, these potential upregulated digenean genes and their associated RNA sequences have not been identified. A further problem that would need to be overcome is the unstable nature of RNA and its tendency to rapidly degrade in the environment [38]. Nevertheless, eRNA assays have been successfully designed to isolate and amplify viral RNA from river and pond water [39] and in future could be used to identify infective fluke stages in the environment.

A sampling strategy based on collecting one sample per habitat visit will likely require optimisation, as the eDNA assay did not always amplify *G. truncatula* eDNA during each sampling visit, where habitats were confirmed to be inhabited by *G. truncatula*. It is suggested that multiple subsampling is needed to increase the sensitivity of eDNA analysis [21, 40], especially when considering that low densities of *G. truncatula* snails on pasture land can lead to significant disease and associated losses [30]. It is acknowledged that eDNA presence in habitats is also likely to have fluctuated across sampling visits. *Galba truncatula* population size and behaviour varies throughout the year [10, 41], whilst miracidia and cercariae are known to favour specific environmental conditions to hatch and shed, respectively [32, 42]. These climatic behavioural differences may lead to variations in levels of DNA released into water by these organisms at certain periods. Subsequent studies would be advised to monitor fluctuations in eDNA detection over time and in conjunction with climatic conditions, as has been done for eDNA of other organisms [43]. Changes in climatic conditions could also affect the time window of measurable eDNA signals in the field, as temperature, sunlight and soil composition are known to affects the rate of DNA degradation [44–48], whilst rainfall could contribute to the dilution or the washing away of potential eDNA signals [43, 49]. The latter factor could in theory lead to reduced specificity for this assay as eDNA signals may be washed to areas of pasture land where the snails do not reside. However, floating metacercariae and *G. truncatula* snails could potentially be washed alongside these eDNA signals and create new fluke infection risk areas [50, 51]. Nevertheless, it would be advisable for future research to screen water sources present on pastures known not to harbour *G. truncatula* snails and fluke infected livestock to confirm assay specificity.

Further eDNA assay development is needed to increase sensitivity. To this end, filter pore size has a major influence on eDNA extract yields, with smaller pore sized filters associated with larger downstream DNA yields [23]. As eDNA strands are estimated to be between 1–10 μm in length on average [52] some eDNA is likely to have escaped the 2.7 μm pore sized filters used for sampling in this study. However, in the laboratory experiment, in each instance where DNA was amplified from 0.7 μm pore sized filters, eDNA was also amplified from the 2.7 μm pore sized filters. Thus, it is assumed that a major proportion of amplifiable *G. truncatula* and digenean eDNA in water will be retained on 2.7 μm pore-sized filters. Using 2.7 μm pore sized filters is advantageous as larger volumes of water can be filtered (prior to clogging), a factor which is associated with increasing eDNA detection probability when species are present in low abundances [22]. As water from typical *G. truncatula* habitats such as bogs, ruts, drainage ditches and stream/pond edges is likely to be shallow and muddy, there may be increased risk of filter clogging during sampling. Clogging occurred in this study where muddy water meant that filtering the specified 500 ml was challenging on some occasions. Prolonged dry weather could also make sampling impossible at certain times in summer if habitats dried out. Nevertheless, water is a requirement for the survival, development and transmission of these digenean parasites and their intermediate snail host [15] and thus, water-based eDNA assay should be capable of analysing most *G. truncatula* habitats during high risk periods for fluke transmission.

Future research is required to realise the promise of eDNA applications. For example, highly sensitive and simple loop-mediated isothermal amplification (LAMP) assays [53–55] should be incorporated into this protocol to detect lower concentrations of target eDNA whilst importantly delivering immediate field relevant data with reduced risk of assay inhibition. LAMP could be used in conjunction with site occupancy models to increase the robustness of presence data further [40]. Furthermore, quantitative PCR assays could be incorporated with the aim of quantifying infection risk if future advancements in measuring eDNA secretion and persistence are achieved [56]. Further transcriptomic and genomic discoveries may further enhance eDNA analysis capabilities allowing for DNA of specific life-cycle stages and genes associated with disease influencing traits (e.g. fluke anthelmintic resistance status, snail infection susceptibility) to be identified in the environment [57–59]. Thus, we propose these technical suggestions as the logical next steps in refining this detection method for supporting fluke control.

## Conclusions

This proof of principle study demonstrates that eDNA assays have the capability of detecting *G. truncatula*, *F. hepatica* and *C. daubneyi* DNA in the environment. Further optimisation of this assay could lead to the development of a test capable of identifying and quantifying *F. hepatica* and *C. daubneyi* infection risk areas on pasture land. This potential eDNA test can be a powerful tool for epidemiological investigations and to improve control strategies for *F. hepatica* and *C. daubneyi* on farms. The general principles of the developed protocols can also be applied to a range of other problematic water inhabiting trematode parasites.

## Abbreviations

CTAB: Cetyltrimethylammonium bromide
eDNA: environmental DNA
FEC: Faecal egg counts
LAMP: Loop-mediated isothermal amplification
PCTE: Polycarbonate track etch
